# Supplementary material for: ‘It felt like a black hole, great uncertainty, but we have to take care for our patients’–Qualitative findings on the effects of the COVID-19 pandemic on specialist palliative home care
Source: PLoS One. 2021 Dec 15;16(12):e0260767. doi: 10.1371/journal.pone.0260767 (PMC8673648; doi:10.1371/journal.pone.0260767)
Supplement: Attachment — Submitted filename: PallPan_focus groups point-by-point reply_fin.docx [file pone.0260767.s001.docx]

|  | Comment | Author’s reply | Changes |
| --- | --- | --- | --- |
|  | Please review your reference list to ensure that it is complete and correct. If you have cited papers that have been retracted, please include the rationale for doing so in the manuscript text, or remove these references and replace them with relevant current references. Any changes to the reference list should be mentioned in the rebuttal letter that accompanies your revised manuscript. If you need to cite a retracted article, indicate the article’s retracted status in the References list and also include a citation and full reference for the retraction notice. | Thank you. No papers were retracted in the meantime. We still added and eliminated some references | Elimination:  Bausewein C, Hodiamont F, Berges N. National Strategy for Palliative Care of severely ill and dying people and their relatives in Pandemics (PallPan) in Germany - Study protocol of a mixed-methods project. (Study protocol did still not receive feedback from journal).  Added references:  Schloesser K, Simon ST, Pauli B, Voltz R, Jung N, Leisse C, u. a. “Saying goodbye all alone with no close support was difficult”- Dying during the COVID-19 pandemic: an online survey among bereaved relatives about end-of-life care for patients with or without SARS-CoV2 infection. BMC Health Services Research. 22. September 2021;21(1):998.  Dos Santos Marques IC, Theiss LM, Johnson CY, McLin E, Ruf BA, Vickers SM, u. a. Implementation of virtual focus groups for qualitative data collection in a global pandemic. The American Journal of Surgery. Mai 2021;221(5):918–22.  Bausewein, Claudia, Simon, Steffen, Forschungsverbund Palliativmedizin im Netzwerk Universitätsmedizin (NUM). Nationale Strategie für die Betreuung von schwerkranken und sterbenden Menschen und ihren Angehörigen in Pandemiezeiten (PallPan) [Internet]. Zenodo; 2021 Juni [zitiert 12. Oktober 2021]. Verfügbar unter: https://zenodo.org/record/5012504  Updated reference:  Mayring P. Qualitative Inhaltsanalyse: Grundlagen und Techniken. 12., überarb. Aufl. Weinheim Basel: Beltz; 2015. 152 S. (from 2010 to 2015 version) |
|  | PallPan is given context throughout, this is in the abstract but the relevance to the study is not clear. Is it needed in the abstract? Please ensure it is defined and clear for an audience not familiar with this project, | Thank you. We have deleted the reference to the PallPan project in the abstract. | Page 2, line 6:  *These patients are at the same time at high risk, if infected, highly constricted by containment measures and dependent on support. ~~PallPan is a national consortium on end of life care in pandemic times.~~* |
|  | For the results, would the authors consider using subheaders? There are not many quotes presented, please consider whether the findings are adequately supported with data | Thank you for this suggestion. We added subheaders in the results section. We also added a table that contains quotes to support our data analysis. | Added subheaders  Added table 3 with quotes. |
|  | I found figure 1 to be confusing and hard to read. Please either provide a clearer version and clarify how it contributes to interpretation of the findings, or remove. | Thank you. We had some technical difficulties when uploading the figure. We have simplified the figure and also made the interconnections in the text more prominent. We believe that the figure is helpful to provide an overview of the key categories and their connections. | Simplified figure.  Added information about interconnections in the manuscript text (see e.g. page 13, line 251-252) |
|  | Either consistently refering to the virus as COVID-19 or SARS-CoV-2 throughout rather than using the terms interchangeably. My recommendation would be to stick with COVID_19 as it is in common usage and recognisable to all sectors of the community. | Thank you for this comment! We have changed all mentions of SARS-CoV-2 to COVID-19. | Changed all mentions of SARS-CoV-2 to COVID-19. |
|  | Careful editing will eliminate the syntax and grammar errors throughout- there are some challenges to both meaning and fluency but they are minimal. | Thank you! We have reviewed the text again and hope to have eliminated all the challenges. | Correction of grammar and syntax error throughout the text. |
|  | Review use of possessive punctuation, for example, it's. | Thank you! We have reviewed the possessive punctuation and hope that we have corrected all errors. | Correction of possessive punctuation |
|  | Review use of brackets and abbreviations- they interfere with fluency. Review use of contractions - for example L238. | Thank you for this comment. We have reviewed the use of brackets and abbreviations, and corrected them, where possible. | L238: *In other regions, every nursing home had its own regulations regarding SPHC teams’ access to nursing home residents.* |
|  | Urgently review use of "quotation marks" it is unclear if you are indicating verbatim quotations from references or direct quotes from the focus group participants or stakeholder interviews. | Thank you! | We have removed all quotation marks. |
|  | L118-125 reads rather like resumes and does not belong in the body of the manuscript. | Thank you for this comment. We included this part to adhere to the COREQ checklist (items 1 to 8) and therefore would keep it in the manuscript. |  |
|  | Table 1: suggest you simplify and remove the focus group composition column and either discuss the composition or include another table. The link to the website you referred to below the table takes you to a blank page with a heading but nothing else. | Thank you! We have added another table with the participants’ characteristics. | Modification of table 1 and addition of table 2.  Added a more detailed description of the sample:  *Most participants were either leading physicians or coordinating nurses (Table 2). In all focus groups, at least nurses and physicians participated. A majority of the participants was female. Teams represented different federal states, and all focus groups where mixed regarding federal states, except for the high incidence group in which only Bavarian teams participated.* |
|  | Figure 1: does not support the inter-connections you discussed in your manuscript- consider re-imagining it to make these connections reflect what you claimed in your results. You might also consider use of an infographic to improve the visual impact of the diagram. | Thank you. We had some technical difficulties when uploading the figure. We have simplified the figure and also made the interconnections in the text more prominent. We believe that the figure is helpful to provide an overview of the key categories and their connections. | Simplified figure.  Added information about interconnections in the manuscript text (see e.g. page 13, line 251-252) |
|  | Method: L 70 what do you mean by consent? | One part of the PallPan study was to conduct a consensus process for the developed strategy and recommendations. As this is not part of the reported study part, we have deleted this part of the sentence. | *This study is part of the PallPan* *study, that aims to develop ~~and consent~~ a national strategy for care of seriously ill patients and their relatives during pandemic times in Germany.* |
|  | You claim to have used qualitative content analysis Philipp (2010- note there is a 2015 version available) but little detail of the process is offered. | Thank you! We have changed the part on data analysis to include more details on the data analysis process. We have also updated the version of the referenced book. | *Changed text on data analysis to: Both focus groups and interviews were analysed using qualitative content analysis (14). Analysis was supported by the software MaxQDA Analytics Pro 2020. Three research questions presented in the focus group guide (pandemic challenges, strategies to handle them, and solutions) guided the analysis. First, the focus group transcripts were paraphrased, and categories were formed inductively from the content. Second, categories were combined to form condensed categories. FN and MJ discussed codes and categories until consensus was reached. Then, we used these categories to code the stakeholder interviews. Codes that did not fit into the existing categories were combined to form new categories. Finally, seven core categories were extracted. FS and MJ discussed the final coding schemes. During the coding, various associations and dependencies between categories emerged, which are outlined in Figure 1.* |
|  | Results: are reported with little original (or translated) text offered - this makes it challenging to discern if the interpretations you made were supported by the data you collected. It would strengthen your analysis considerably to support it with quotes from at least a couple of different participants/stakeholders otherwise it is more of a summary than a qualitative analysis. | Thank you for the suggestion to add original text. We have added a quote for every category to support our interpretation. Quotes are presented in form of a table, because quotes were often associated with more than one category. | Added table 3: Quotes and categories. |
|  | Consider more clearly outlining the recommendations for future practice as these will be of interest to providers. | Thank you. We have added another reference to the recommendations that were developed in the PallPan-Project. | Added text (page 18, line 353-353) |
|  | * Please review and correct punctuation used in the title - it appears that a comma is used at the beginning rather than an opening quotation/speech mark (this should correspond with the quotation/speech mark that is correctly used at the end of the quotation). | Thank you, we have deleted the comma and used a quotation mark. |  |
|  | * Abstract - it seems inconsistent that there is no heading for the first (background/introduction) part of the abstract, but then there are headings for Aim/Method/Results/Discussion. Suggest adding a heading for the first section for consistency. | Thank you. We have added a heading to this section | Added heading “background” at the beginning of the abstract. |
|  | * use of COREQ reporting guidelines/checklist - I could not see any mention of COREQ checklist Item 28 - whether the study participants provided feedback on the findings? If not, were they offered a reasonable opportunity to do so? Please address this as per COREQ. | Thank you for raising this important point! The teams were invited to an online meeting, where the results of the study were presented to them, and they were asked to provide feedback. | Added text (page 7, 138-140): After both study parts (focus groups/interviews and questionnaire) were completed, all participants were invited to an online meeting, in which the results of the study were presented to them, and they were asked to provide feedback. |
|  | * Results - Is "Between September 15, 2020 and September 29, 2020, four focus groups with 4-5 participants were conducted" really a result of the study? I would suggest that the conduct of these FGs is more accurately a research method of data collection. As such, I think this sentence should be relocated to the appropriate ,methods/data collection section - rather than being reported here as a 'result' of the study. | Thank you for this comment that we carefully considered. We believe it is common to place the time of data collection in the results section. Furthermore, because of the dynamic progression of the pandemic, the timing of the data assessment directly influences the results and discussion. We therefore considered this information as adequately placed in the results section. |  |
|  | * Limitations - Reference #21 - cited in support of using online FGs: I note this supporting reference comes from the 'Journal of Advertising' wonder why this is considered appropriate? I suggest that you could find a much more relevant source to cite here in this (health research) context. | Thank you for providing such an excellent reference. When the manuscript was first written, there was not much information available regarding the use of virtual focus groups. We have added the reference you suggested. | Additional reference:  Dos Santos Marques IC, Theiss LM, Johnson CY, McLin E, Ruf BA, Vickers SM, u. a. Implementation of virtual focus groups for qualitative data collection in a global pandemic. The American Journal of Surgery. Mai 2021;221(5):918–22. |
